# Sodium Chloride Induced Stress Responses of Antioxidative Activities in Leaves and Roots of Pistachio Rootstock

**DOI:** 10.3390/biom10020189

**Published:** 2020-01-26

**Authors:** Mohammad Akbari, Ramesh Katam, Rabab Husain, Mostafa Farajpour, Silvia Mazzuca, Nasser Mahna

**Affiliations:** 1 Department of Horticultural Sciences, University of Tabriz, Tabriz 51666, Iran; hort.moh@gmail.com; 2 Department of Biological Sciences, Florida A&M University, Tallahassee, FL 32307, USA; rqh5279@psu.edu; 3 School of Science, Engineering, and Technology, Pennsylvania State University, Harrisburg, PA 17057, USA; 4 Seed and Plant Improvement Research Department, Mazandaran Agricultural and Natural Resources Research and Education Center, Agricultural Research, Education and Extension Organization (AREEO), Sari 19395-1113, Iran; farajpour_m@alumni.ut.ac.ir; 5 Dipartimento di Chimica e Tecnologie Chimiche, Universita Della, Calabria, 87036 Rende, Italy; silvia.mazzuca@unical.it

**Keywords:** antioxidant enzymes, lipid peroxidation, NaCl, *Pistacia vera*, rootstock, salinity stress

## Abstract

Salinity substantially affects plant growth and crop productivity worldwide. Plants adopt several biochemical mechanisms including regulation of antioxidant biosynthesis to protect themselves against the toxic effects induced by the stress. One-year-old pistachio rootstock exhibiting different degrees of salinity tolerance were subjected to sodium chloride induced stress to identify genetic diversity among cultivated pistachio rootstock for their antioxidant responses, and to determine the correlation of these enzymes to salinity stress. Leaves and roots were harvested following NaCl-induced stress. The results showed that a higher concentration of NaCl treatment induced oxidative stress in the leaf tissue and to a lesser extent in the roots. Both tissues showed an increase in ascorbate peroxidase, superoxide dismutase, catalase, glutathione reductase, peroxidase, and malondialdehyde. Responses of antioxidant enzymes were cultivar dependent, as well as temporal and dependent on the salinity level. Linear and quadratic regression model analysis revealed significant correlation of enzyme activities to salinity treatment in both tissues. The variation in salinity tolerance reflected their capabilities in orchestrating antioxidant enzymes at the roots and harmonized across the cell membranes of the leaves. This study provides a better understanding of root and leaf coordination in regulating the antioxidant enzymes to NaCl induced oxidative stress.

## 1. Introduction

Pistachio (*Pistacia vera* L. Anacardiaceae) is a widely cultivated and important tree nut crop. These nuts provide rich sources of health promoting nutrients such as proteins, phenols, antioxidants, and minerals [1]. The crop is grown in semi-arid regions where high soil salinity has adversely affected the pistachio cultivation and production. Soil salinization is a serious menace for crop cultivation, productivity, and has devastating global effects, estimated at 50% of land loss by 2050, affecting the crop sustainability and food security [2]. Salinity affects the crop productivity through various physiological changes resulting in osmotic stress by making it harder for roots to absorb water, causing internal dehydration, and ion toxicity caused by the direct accumulation of salts. The ionic imbalance and hyperosmotic stress resulting from salinity manifest as oxidative stress and salinity-induced accumulation of Na, which competes with K ions, causing inhibition of metabolic enzymes [3,4]. The cascade of oxidative reactions causes the inactivation of enzymes and protein degradation. These reactions damage the cell membranes, photosynthetic pigments, proteins, nucleic acids, and lipids. Thus, it is critical for plants to regulate the ROS levels in the cells [5].

Several studies suggest that the activity of antioxidant enzymes is correlated with plant tolerance to salinity [6]. In general, plants are fortified with different classes of protective and restitution systems to minimize the adverse effects of oxidative damage. The first order class enzymes are the scavenging ROS enzymes such as ascorbate peroxidase (APX), peroxidase (POD), catalase (CAT), superoxide dismutase (SOD), and the lipid peroxidation detoxification products malondialdehyde (MDA). Additionally, a class of low molecular mass antioxidants, which regenerate the oxidized antioxidants and turn them into their active forms, namely, ascorbate, glutathione, and glutathione reductase (GR) are associated with the protective mechanism [7]. Briefly, SOD acts as the first line antioxidant systems of plants, catalyzing the dismutation of the O_2_^−^ to O_2_ and H_2_O_2_, thus causing cell damage [8]. Often, PODs are used as markers in physiological studies and their activity level is used as an index for evaluating oxidative stress. POD is well acknowledged as a stress enzyme in plants, oxidizing numerous substrates using H_2_O_2_ and hampering the excess agglomeration of H_2_O_2,_ generated by normal metabolism or stress conditions [9]. 

PODs catalyze the oxido-reduction between H_2_O_2_ and various reductants and enhance the plant tolerance to salinity [10]. Catalases, commonly located in peroxisomes, catalyze H_2_O_2_ to water and oxygen and are involved in photorespiration [11]. The most important H_2_O_2_ detoxification pathway is the ascorbate-glutathione system involving APX and GR enzymes, occurring in chloroplasts, cytosol, mitochondria, and peroxisomes. APX uses two molecules of ascorbate as a specific electron donor for reducing H_2_O_2_ to water [12]. Subsequently, the oxidized ascorbate is regenerated by monodehydro ascorbate reductase or dehydro ascorbate reductase using reduced glutathione (GSH), generating glutathione disulfide (GSSG), which in turn is reduced to two molecules of GSH with the aid of NADPH as the electron donor catalyzed by the enzyme GR [13]. Furthermore, ROS are able to affect the antioxidative enzyme activities leading to lipid peroxidation and MDA formation. The content of MDA, the main byproduct of lipid peroxidation, has commonly been considered as a biomarker of cell membrane damage in plants [14].

Thus, the relatively quantitative changes of these enzymes and their correlation analyses play a major role in determining the plant tolerance to the salinity stress, suggesting they can be applied as biochemical markers to select a more tolerant plant or to investigate the level of salinity tolerance in plants [15]. Very few studies are available on the differences in oxidative stress and antioxidative defenses in different organs and prolonged periods of stress. Pistachios are relatively better adapted to salinity as compared with most tree nut crops [16]. Therefore, evaluating genetic diversity among pistachio germplasm for their salt tolerance could provide a better understanding of salt tolerance in pistachio genotypes, especially rootstock, for introducing potential traits in a breeding program [17]. Correlating studies of the changes in these enzymes in different cultivars are necessary to determine the biochemical pathways involved in regulating various degrees of salt stress responses in pistachio. Hence, the aims of this study include the following: (1) investigate the tissue-specific responses of the antioxidant enzymes associated with salinity in the leaf and root tissues, (2) elucidate biochemical modification mechanisms for salt tolerance, and (3) establish suitable correlation and regression models to explain variability in salt tolerance among various rootstock. To our knowledge, this is the first report of a genetic variation studies on temporal antioxidant responses in root and leaf pistachio rootstock in response to varying NaCl-mediated stresses.

## 2. Results

### 2.1. The Effect of Salinity on Plant Growth and Morphology

After 100 days of salt treatments at 8 dS/m concentration, rootstock did not show any symptoms. However, at 12 dS/m NaCl, leaf tip necrosis was observed in Akbari and Kale-Ghouchi (KG) rootstock, and up to 50% of leaves showed senescence symptoms and desiccation at 16 dS/m NaCl treatment. Rootstock Ghazvini, followed by Badami and UCB-1 developed leaf tip necrosis at 16 dS/m. We further investigated the enzyme activities (SOD, POD, CAT, APX, and GR) and MDA content of five pistachio rootstock in leaf tissues at different salinity levels and measured at different time courses (25 to 100 days), whereas for roots the activities were measured at 100 days of treatment and compared with enzyme activities in leaf tissues. Results of these enzyme activities and the MDA content are shown below.

### 2.2. Antioxidant Enzyme Activity

**Figure 1:**
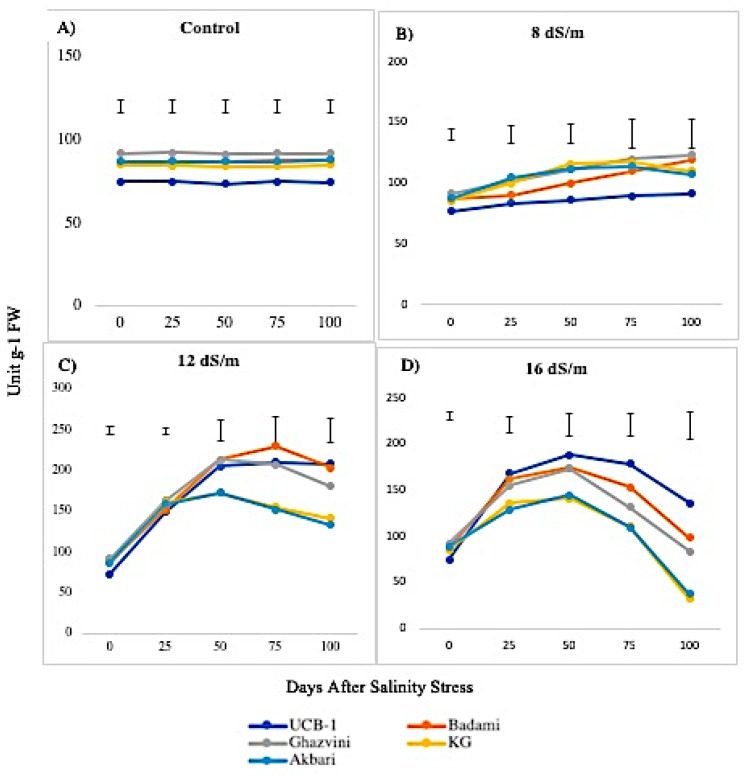
Effect of NaCl-mediated stress on SOD activity in leaves of pistachio.

**Figure 2:**
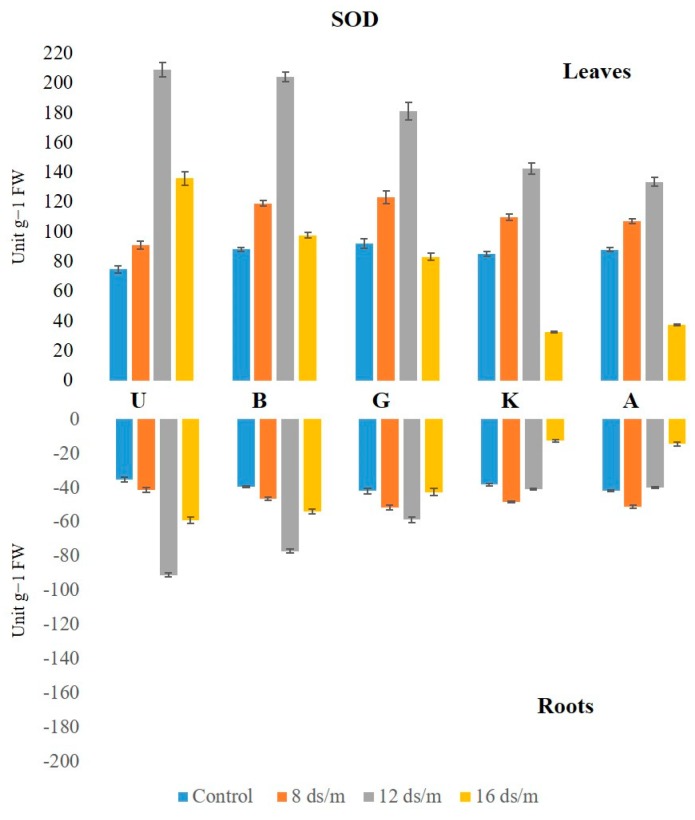
Effect of NaCl-mediated stress at 100 days of treatment on SOD activity in leaves and roots of pistachio.

The SOD activity was higher in leaves than in the roots both under normal and saline conditions. Under control conditions, the activity of SOD in leaf remained constant over time under control conditions, with the highest being in Ghazvini rootstock followed by Badami, Akbari, KG, and UCB-1 (Figure 1). Leaf tissue displayed significantly higher SOD activity relative to NaCl treatments in all rootstock. NaCl at 8 dS/m induced SOD activity in all cultivars. At 12 dS/m, it had significantly elevated SOD levels in rootstock UCB-1 (2.8-fold) followed by Badami, Ghazvini, KG, and Akbari in decreasing order. At 16 dS/m NaCl concentration, increased SOD activity was noticed until 50 days, gradually declining in all rootstock thereafter, with the highest activity being recorded in UCB-1, followed by Badami, Ghazvini, Akbari, and KG. In leaf tissue, the highest SOD activity was 209 in 12 dS/m in UCB-1, which decreased to 135.8 in 16 dS/m. In root tissue, the enzyme activity increased in salt treated samples, and, at day 100, the maximum amount of SOD was observed in UCB-1 at 12 dS/m (91.1), decreasing at 16 dS/m (59.07) (Figure 2).

**Figure 3:**
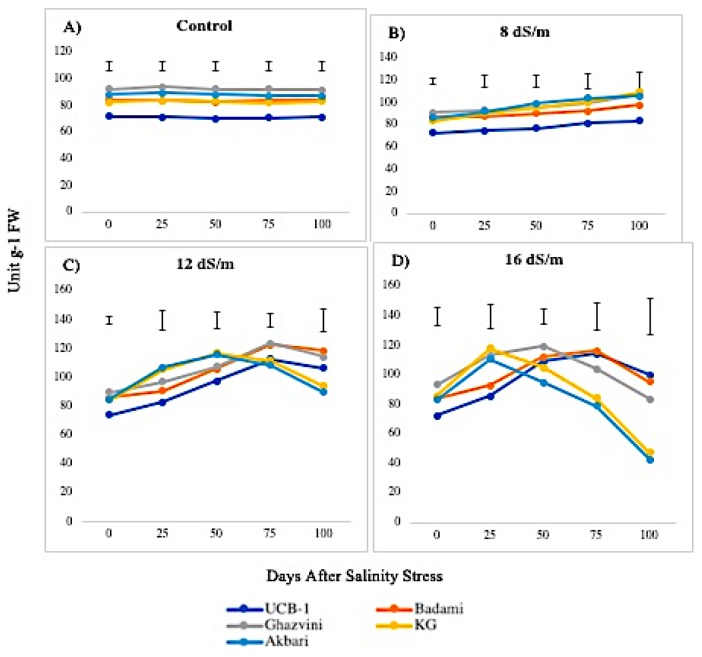
Effect of NaCl-mediated stress on POD activity in leaves of pistachio.

**Figure 4:**
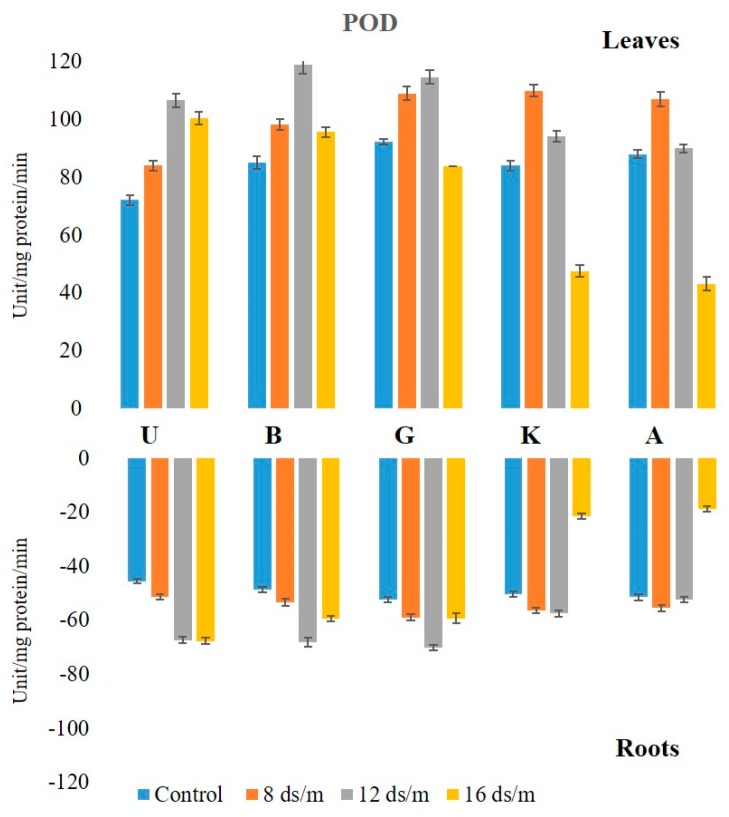
Effect of NaCl-mediated stress at 100 days of treatment on POD activity in leaves and roots of pistachio.

The POD levels were higher in control leaf tissues than in root tissues and displayed significant elevations in both leaves and roots in response to NaCl treatments. Under control conditions, the POD activity levels remained unchanged in all rootstock in both tissues in descending order of UCB-1, followed by KG, Akbari, Badami, and Ghazvini. At 8 dS/m NaCl concentration, all cultivars showed increased POD activity through 100 days in leaf tissue (Figure 3). At 12 dS/m, the activity increased up to 75 days in all cultivars, except in Akbari and KG, where a decline occurred at day 50. Under 16 dS/m NaCl treatment, the decline in enzyme activity started at day 50 in KG and Akbari, while in UCB-1, Ghazvini, and Badami, the activity declined at day 75. Among the rootstock, UCB-1 maintained higher enzymatic activity (1.4-fold) after 100 days. The maximum POD level in the root was observed in rootstock UCB-1 (1.5-fold), at 16 dS/m salinity treatment, as compared with the control (Figure 4). For POD, in both tissues, UCB-1 showed the highest activity and Akbari the lowest after 100 days. However, in the controls, UCB-1 showed the lowest values in both tissues, and, during stress, they increased to the highest POD values in both tissues.

**Figure 5:**
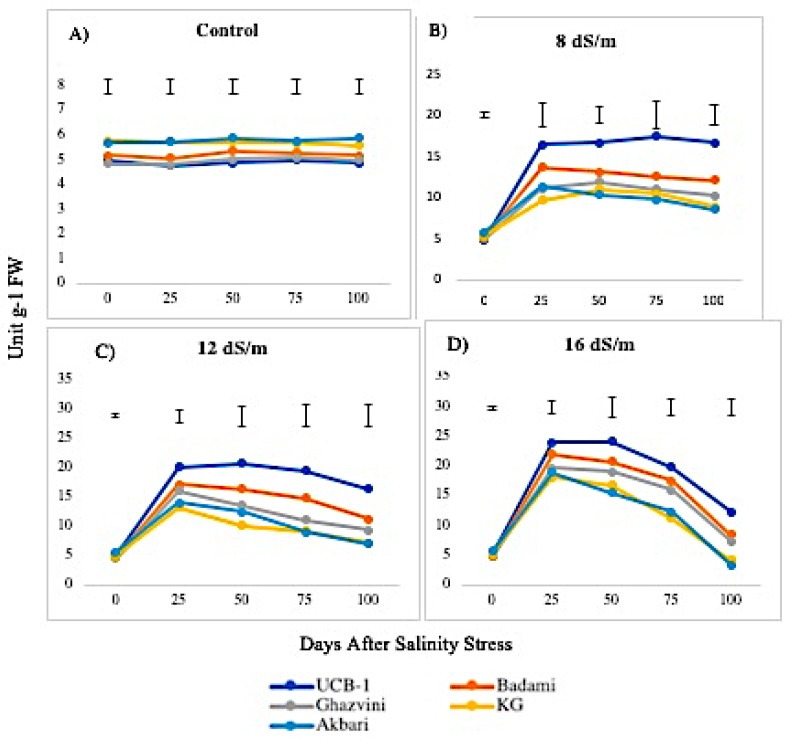
Effect of NaCl-mediated stress on CAT activity in leaves of pistachio.

**Figure 6:**
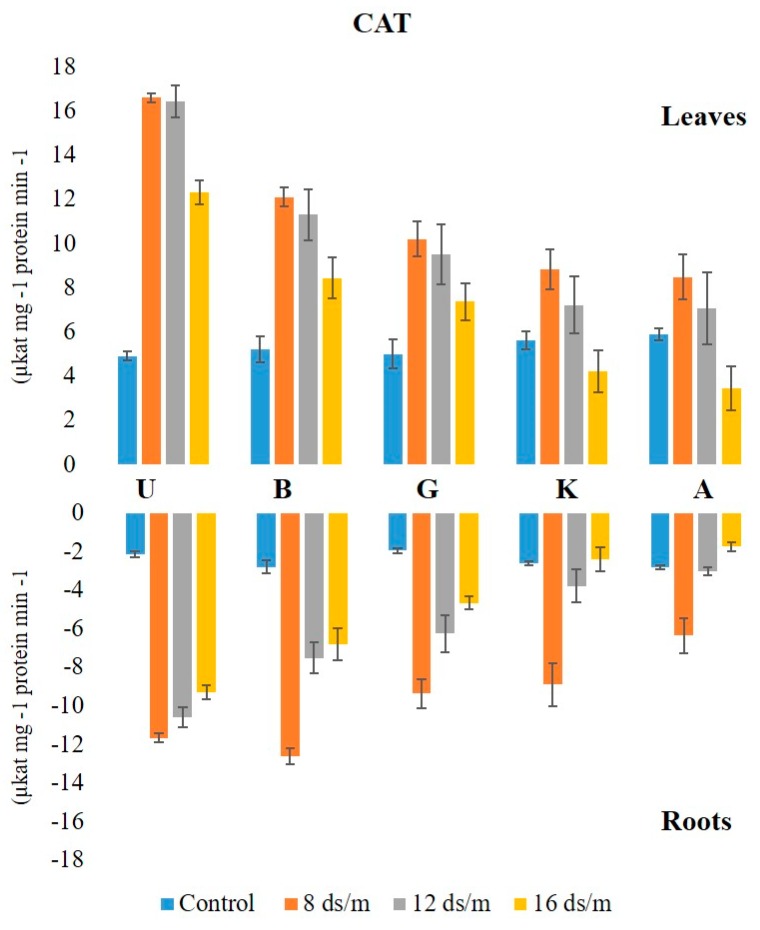
Effect of NaCl-mediated stress at 100 days of treatment on CAT activity in leaves and roots of pistachio.

The activity of CAT was higher in leaf than in the root under control conditions in all rootstock. No significant change in CAT activity was observed in control leaf tissues over time, with Akbari being the highest, followed by KG, Badami, Ghazvini, and UCB-1. At both 8 dS/m and 12 dS/m, the activities increased at day 25 and remained relatively stable afterwards (Figure 5). However, at 16 dS/m, the activity increased at day 25, showing a gradual decline in all rootstock thereafter. The maximum level of CAT was observed in the leaves of rootstock UCB-1 at 25 days under 16 dS/m saline treatment (5-fold), decreasing thereafter. In root tissue, the maximum CAT enzyme amount was observed in Badami at NaCl 8 dS/m (12.61), which further decreased at 12 dS/m and 16 dS/m. At 16 dS/m, the maximum CAT level was observed in rootstock UCB-1 (9.29), followed by Badami, Ghazvini, KG, and Akbari (Figure 6). 

**Figure 7:**
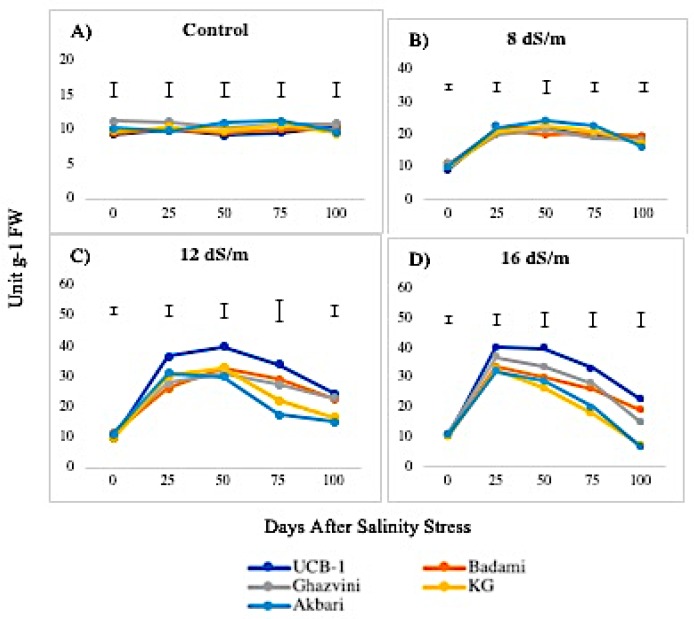
Effect of NaCl-mediated stress on APX activity in pistachio leaf.

**Figure 8:**
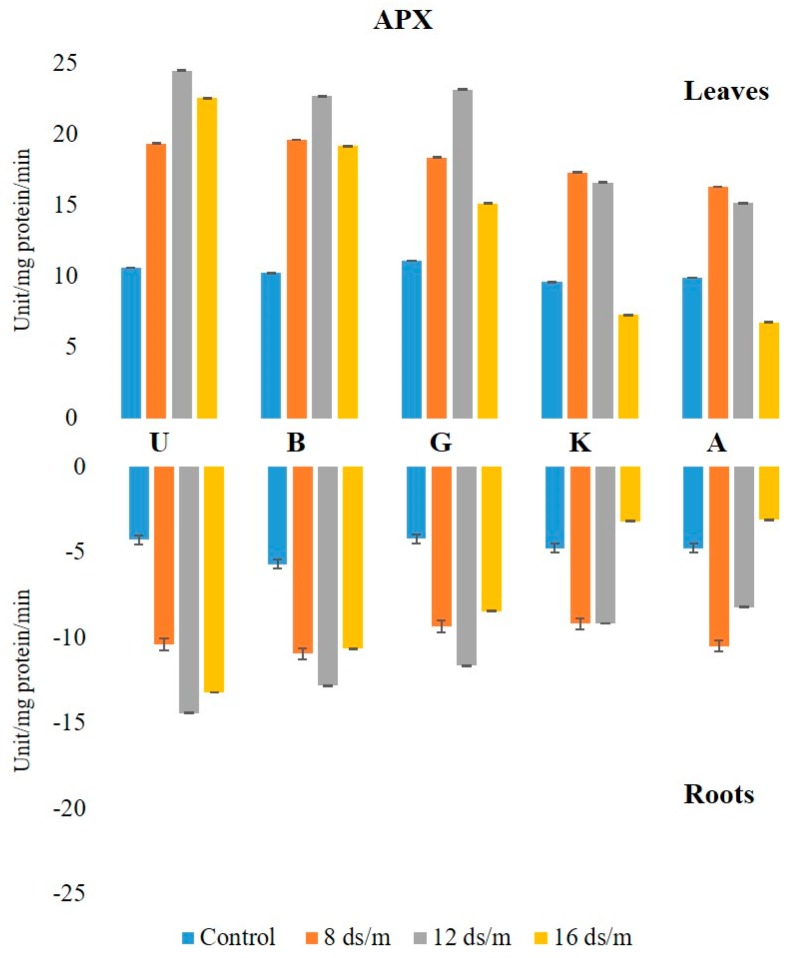
Effect of NaCl-mediated stress at 100 days of treatment on APX activity in leaves and roots of pistachio.

The activity of APX was higher in leaves than in roots, both in controls and in saline conditions. The APX activity in the leaves under the control condition was in the following decreasing order: Ghazvini followed by UCB-1, Badami, Akbari, and KG, remaining relatively unchanged over 100 days, and then significantly increased due to salinity (Figure 7). At 8 dS/m, 12 dS/m, and 16 dS/m NaCl treatments, all leaf tissues in cultivars showed a significant increase in APX activity up to 50 days and later declined gradually, with UCB-1 being the highest, followed by Badami, Gahzvini, KG, and Akbari. In root tissues, APX exhibited a significant increase due to salinity at 8 dS/m, 12 dS/m, and 16 dS/m in all the cultivars except in KG and Akbari. At 16 dS/m, UCB-1 was the highest, followed by Badami, Ghazvini, KG, and Akbari (Figure 8).

**Figure 9:**
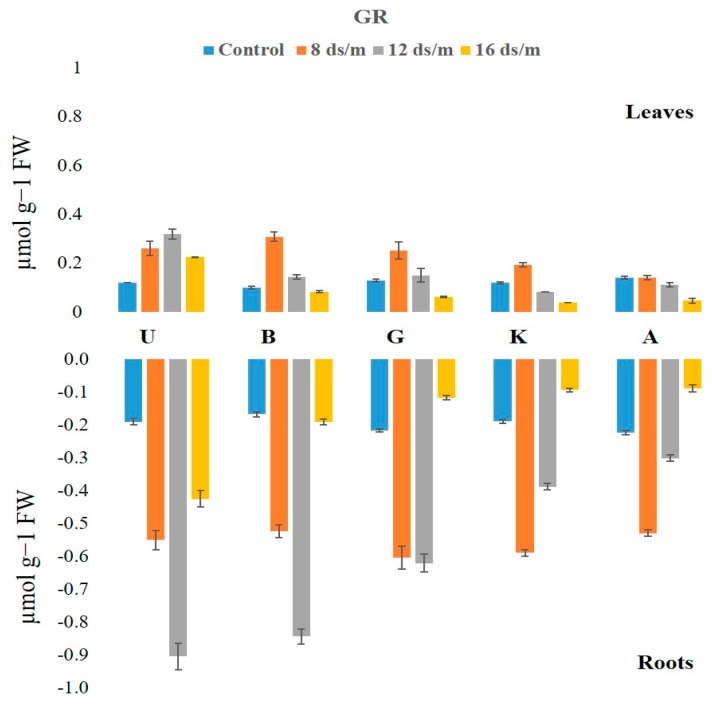
Effect of NaCl-mediated stress on GR activity in root tissues of pistachio.

**Figure 10:**
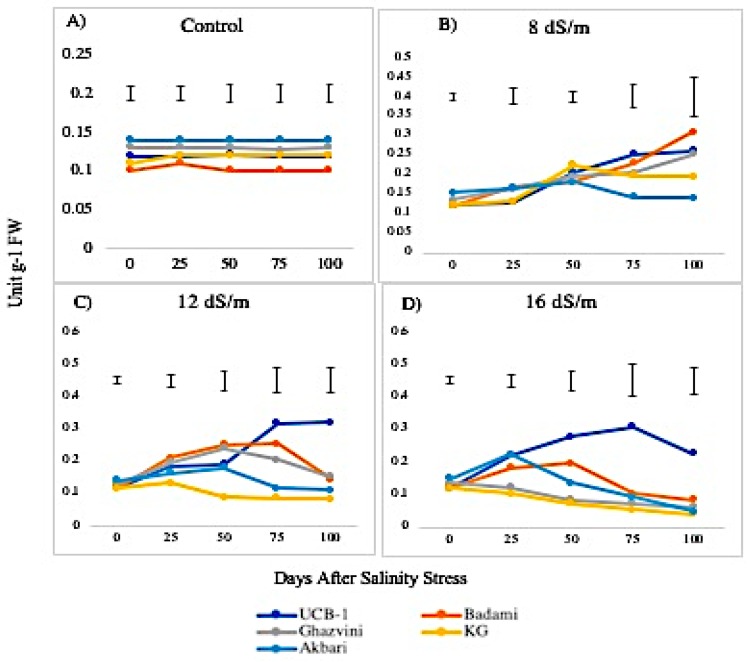
Effect of NaCl-mediated stress at 100 days of treatment on GR activity in leaves of pistachio.

Glutathione reductase (GR) activity exhibited significant changes in leaf and root tissues (Figure 9). The GR activity was relatively low in leaves as compared with roots, under control conditions in the following decreasing order: Akbari followed by Ghazvini, UCB-1, KG, and Badami. At low concentrations of salt (8 dS/m) the GR levels elevated in all cultivars in both tissues. At moderate and high salt stress (12 dS/m and 16 dS/m), the GR activity in leaves showed mixed responses among the cultivars (Figure 10). For example, UCB-1 showed a linear increase in GR level at NaCl 12 dS/m and reduced at 16 dS/m in leaf tissue, while all other rootstock showed a gradual decrease in GR activity in both tissues. Under the 16 dS/m saline treatment, the maximum increase in the amount of GR activity was observed in the UCB-1 cultivar (1.9-fold in leaves and 2.2-fold in roots), followed by rootstock Badami, Ghazvini, KG, and Akbari in decreasing order. 

### 2.3. Lipid Peroxidation

**Figure 11:**
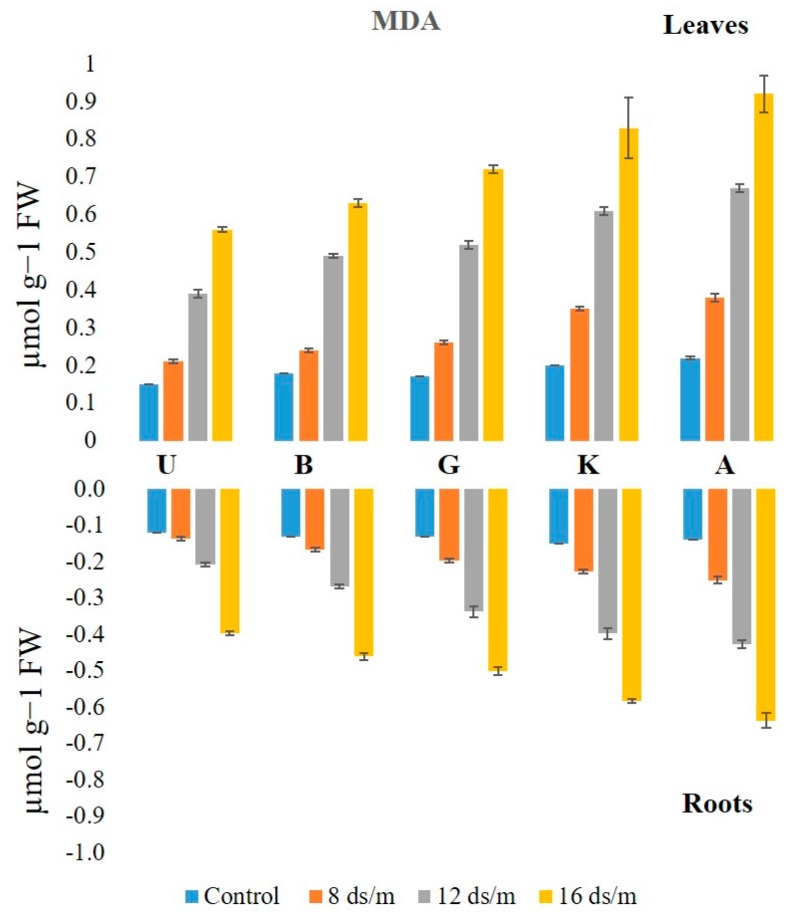
Effect of NaCl-mediated stress on MDA activity in roots of pistachio.

**Figure 12:**
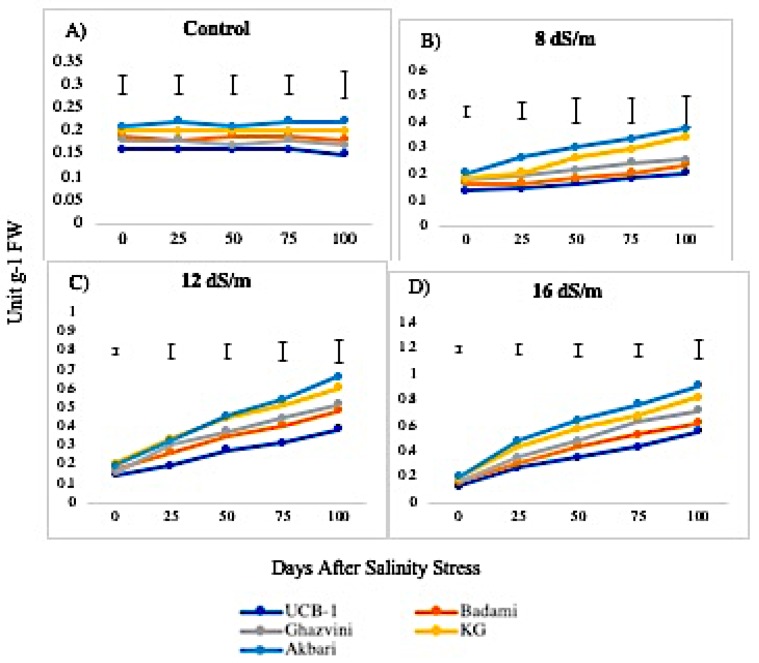
Effect of NaCl-mediated at 100 days of treatment on MDA activity in leaves of pistachio at different time intervals.

Lipid peroxidation levels, measured from MDA concentration, were higher in leaves than in roots under control conditions (Figure 11). The MDA levels increased linearly from low to high levels of NaCl in all rootstock, generally being higher in leaf as compared to root tissues. In leaf tissue, the level of MDA measured after 100 days of treatment with NaCl 16 dS/m was the highest in Akbari in both tissues (4.1-fold in leaves and 4.5-fold in roots) followed by KG, Ghazvini, Badami, and UCB-1 (Figure 12). A similar trend of MDA activity was observed in roots to NaCl treatments 8, 12, and 16 dS/m, with the maximum being recorded in rootstock Akbari followed by KG, Ghazvini, Badami, and UCB-1 (Figure 11).

### 2.4. Correlation Analysis

Four different correlation coefficients between the measured characters were investigated in leaves, roots, leaves with roots, and roots with leaves (Table 1).

All measured characters had a significant correlation with each other in leaves with roots and roots with leaves (except CAT in leaf with POD in root). The correlation coefficient of MDA content between leaves and roots was one. The MDA content in leaves was negatively correlated with all the other enzymes in leaves, and its correlation coefficients reached up to −0.99 (*p* < 0.01) with APX. A similar result was found in the root, as the MDA was negatively correlated with other studied characters in both roots and leaves. All correlation coefficients of the MDA contents in leaves were negatively correlated with the others in the roots. The maximum and minimum correlation coefficients between the enzymes in leaves were found between APX and MDA (−0.99), and CAT and SOD (0.29), respectively. The highest positive correlation coefficient in root enzymes was observed between APX and SOD (0.98), whereas the highest negative correlation coefficient was observed between MDA and GR (−0.99). According to the results, in both root and leaf, the SOD, POD, and APX were significant enzymes involved in salinity tolerance. Data were analyzed as an unbalanced complete randomized design (CRD) between two different groups of rootstocks with tolerant (UCB-1, Badami, Ghazvini) and susceptible (Akbari and KG) showing that SOD, POD, and APX were the most effective indices, respectively (Table 2).

### 2.5. Model Analysis

**Figure 13:**
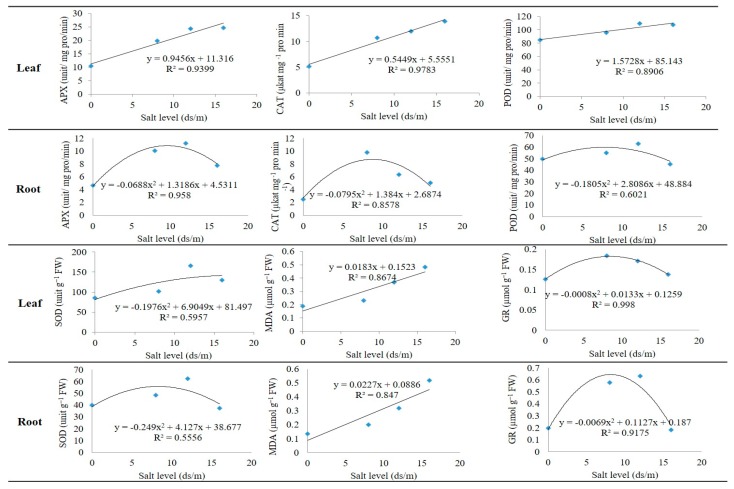
Linear and quadratic models of SOD, POD, CAT, APX, GRT, and MDA indices in leaves and roots of pistachio rootstock.

The linear and quadratic regression models for the enzyme activities of pistachio rootstock, in both leaves and roots, were evaluated. The enzymes APX, CAT, POD, and MDA had a linear regression model in leaves while the enzymes SOD and GR had a quadratic regression model with the salinity levels (Figure 13). In this study, nine of the 12 models had a high R-square. The three leaf enzymes, namely APX, CAT, and POD, had a linear regression model, but a quadratic regression model in roots. Among these models, CAT in leaves had the highest R-square (97%). Therefore, by increasing the salinity level, the concentration of the APX, CAT, and POD, enzymes in the leaves, as well as MDA in both leaves and roots, increased. The SOD and GR enzymes had quadratic regression models in both roots and leaves. In all of the models, GR in leaves had the highest R-square and explained more than 99% of the variance of the data, whereas the model of SOD in root only explained about 55% of the variation. The MDA had a linear regression model with a high coefficient of determination in both leaves and roots.

## 3. Discussion

Salt stress results in the accumulation of ROS due to changes in the electron transport chain and induces protection mechanisms during salt stress [18]. On the one hand, higher salinity soil conditions reportedly induce oxidative stress in plant organelles [19]. On the other hand, overexpression of antioxidant enzymes is tissue associated with improvement in salt tolerance [20].

### 3.1. Quantitative Variation among the Antioxidative Enzymes Is Tissue Specific and Depends on Both Duration and Levels of NaCl-Induced Salt Stress

In our studies under normal irrigation conditions, antioxidative enzyme levels of SOD, POD, CAT, and APX and lipid peroxidase activity were maintained higher in leaf tissues than in root tissues in all rootstock. On the contrary, GR showed lower levels in leaf tissues as compared with root tissues. Low (8 dS/m) and moderate (12 dS/m) salt stress elevated the SOD, POD, and CAT levels up to a 100 day stress period in leaf tissues, while, at higher salt levels (16 dS/m), their activity was maintained for a 75 day stress treatment, beyond that, it declined in all rootstock. However, APX activity was elevated only up to a 50 day stress duration. Both in root and leaf tissues, these enzyme levels were elevated at a 100 day stress duration at low and moderate stress levels, while the levels declined at higher salinity stress. Salt tolerant pistachio rootstock showed lower levels of enzyme activities as compared with their levels in susceptible cultivars under normal growth conditions, however, upon the stress, tolerant rootstock exhibited higher levels over the susceptible tissues.

One of the enzymes, SOD, plays a major role in ROS scavenging in plants and is considered as the first line of defense against the toxic effects of elevated ROS levels [21,22]. SOD catalyzes the dismutation of superoxide radicals to H_2_O_2_ and O_2_. The increase of SOD activity might be the reason for enhanced O_2_ generation, as a result of electron leakage from the electron transport chains to molecular oxygen [23]. Similarly, POD activity was significantly altered in both tissues after salt treatments. POD levels were higher in control leaf tissues than in root tissues, produced by POD, CAT, APX, and other antioxidant enzymes. SOD levels were higher in control leaf tissues as compared with root tissues, and SOD levels were significantly higher in both leaf and root tissues due to salinity treatments. No significant difference was observed between the control and 8 dSm^−1^ in the leaf tissue, but a significant difference was observed in the root tissues at a low salt concentration (8 dSm^−1^) and also at increasing salinity levels of 12 dSm^−1^ and 16 dSm^−1^.

The difference was significant in the root tissue as compared with the increased SOD in leaf tissue, suggesting SOD activity in roots was insensitive to low salinity and seems to be organ specific. By increasing salinity to 12 dSm^−1^ and 16 dSm^−1^, the rootstock of UCB-1, Badami, and Ghazvini exhibited higher SOD activity to overcome the oxidative stress as compared with KG and Akbari rootstock in both tissues. Our previous studies on osmoregulation showed that the rootstock UCB-1, Badami, and Ghazvini are relatively salt tolerant as compared with KG and Akbari [24]. Although SOD levels were low in tolerant rootstock, their increase in enzymatic activities was higher in response to salinity stress in relatively tolerant rootstock, suggesting their O_2_^−^ scavenging ability. A similar increase in SOD activity in tolerant genotypes has been observed in cotton in response to saline stress [25].

POD is the primary enzyme and the increase in its activity detoxifies H_2_O_2_ in chloroplast and cytosol during oxidative stress [26,27]. POD levels have been increased in tolerant rootstock (UCB-1 and Ghazvani) to high saline stress in both tissues, while the activity decreased in less tolerant rootstock (Badami, KG, and Akbari). POD activity has been reported to be high in salt tolerant cotton cultivar under salt stress, while the enzyme activity remained constant as compared with the control [28]. Low levels of salinity (8 dS/m, and 12 dS/m) for prolonged periods induced higher activities of SOD and POD in the leaf tissue, while the higher salt concentration increased the enzyme activity for 25 to 50 days, reducing significantly thereafter. Rootstock UCB-1 maintained a higher enzymatic activity in both tissues (1.4-fold in leaf and 1.5-fold in root) during stress over 100 days of treatment as compared with other cultivars in both tissues at 16 dS/m salinity treatment. It is noteworthy that in both tissues, the POD activities were similar to the SOD in the rootstock in the following order: UCB-1 followed by Badami, Ghazvini, KG, and Akbari. POD activity in both tissues was the highest in UCB-1, and the lowest in Akbari, while the opposite results were seen in their controls. 

CAT plays an important role in the antioxidant system. Both POD and CAT constitute a main H_2_O_2_ scavenging system in the cells [29]. The present data showed that CAT activity was higher in the leaves than in the roots under both saline and control conditions, however, the rate of increase in enzyme activity to salt stress is relatively high in roots (three to four times) as compared with leaf tissues among all the rootstock. Compared to the controls, the activity of CAT in the roots increased more than that in the leaves. Although the control CAT activity in leaf and root was relatively higher in Akbari as compared with UCB-1, at 16 dS/m, UCB-1 showed the highest CAT activity, whereas the Akbari performed the lowest among all the rootstock. CAT is involved in salinity stress and increases the resistance of plants to saline conditions [19,30]. CAT activity significantly increased in response to salinity.

Furthermore, CAT activity against salt-induced oxidative stress appeared to be organ specific, as well as cultivar and the duration of stress duration dependent. Increased CAT activity was recorded in leaf tissues of tolerant rootstock (UCB-1, Badami, and Ghazvini) up to 100 days at high NaCl stress. However, in susceptible rootstock (Akbari and KG), the amount of CAT decreased, as compared with the control. Similar findings have been reported that CAT activity has been increased gradually by increasing the salt treatments in the salt tolerant maize cultivars and reduced significantly in the salt-sensitive ones [31]. Concomitant with the results obtained here, salt-tolerant rice genotypes had shown significantly higher CAT activity as compared with that of susceptible genotypes [19]. At high salinity, the APX activity was elevated in the tolerant rootstock of UCB-1, Ghazvini, and Badami in leaves, whereas it decreased in less tolerant rootstock, Akbari, and KG, suggesting a stronger correlation of APX activity to salt tolerance. Higher APX activity has been observed in salt tolerant genotypes as compared with their salt sensitive counterparts, in different tissues [12,32]. Genetic engineering of plum plants by cytosolic APX gene enhanced the tolerance to salt stress in in vitro plum plants [33]. Accordingly, overexpression of cytosolic APX in tomato has been reported to confer tolerance to salt stress [34].

GR, belonging to the family of NADPH-dependent oxidoreductase, plays a key role in cell defense against ROS through maintaining the reduced GSH pool at a cellular level by catalyzing the reduction of GSSG (oxidized glutathione) to GSH. In this study, the GR activity was higher in the roots than in the leaves under both saline and normal growth conditions. Salinity stress significantly enhanced GR activity in both leaves and roots, indicating its role in scavenging H_2_O_2_ formed as a result of increasing SOD activity under salinity stress. After 100 days of salinity stress, the salt tolerant rootstock, UCB-1, showed a remarkable increase in GR enzyme in root tissue. It has been reported that the salt-induced increase in GR activity is higher in salt-tolerant rice genotypes as compared with salt-sensitive rice genotypes [35].

### 3.2. Lipid Peroxidation Activity in Relation to Antioxidant Enzymes among Rootstock

MDA is the final product of lipid peroxidation that has attracted widespread interest as an indicator of damage to the cell membrane system. High levels of ROS in the cells cause membrane lipid peroxidation. The MDA content was increased due to salinity stress (at 16 dSm^−1^) in leaf and root tissues of the rootstock. As compared with the control group, the MDA increments in the leaves of pistachio rootstock were higher than those in the roots, indicating that the leaves were more affected by the salinity induced oxidative damage, most likely due to the increased activity of enzymatic and non-enzymatic pathways detoxifying ROS. Similarly, in lentil, the shoot and root responses to salinity stress indicated that roots are less affected by the salinity-induced oxidative stress [36]. In another report, lipid peroxidation has been only observed in the roots of *Crithmum maritimum* L. under high salinity, while the leaves were not seriously influenced by salinity stress [37]. In this study, susceptible rootstock Akbari had the maximum MDA content in the leaves and roots and the minimum MDA content in leaves and roots was observed in tolerant rootstock UCB-1 exposed to high stress. Unaffected MDA content suggests reduced membrane damage, which indicates the superior ability of UCB-1 over the other studied rootstock to cope with the saline conditions. The relationship between MDA content and enzyme activity shows that at the 75 day higher NaCl treatment (16 dSm^−1^), the activity would increase in all rootstock. This is likely due to H_2_O_2_ accumulation, which causes lipid peroxidation causing an increase in MDA concentration. This increase in MDA damaged the membrane of cells causing up to a 50 percent leaf loss in Akbari and KG.

### 3.3. Correlation between H_2_O_2_ Activity and Antioxidant Responses

A positive correlation was observed between SOD and CAT in roots but not in leaves, while SOD activity correlated well with APX activity in both leaves and roots (Table 1). It has been suggested that both CAT and APX, which are responsible for detoxification of H_2_O_2_, are equally important [38] in this process. Although the CAT activity significantly increased, APX activity increased even more. In tomato, CAT activity is not associated with significant changes in the leaves, while significant changes have been observed in the roots [39]. Similar results have been observed in CAT with decreased activity under stress situations as compared with the control in the leaf tissue of cowpea [40]. The activities of CAT and APX enzymes changed depending on the time of salt treatment and salt concentration and were highly correlated in the leaves (0.97) and roots (0.99), suggesting that both enzymes are essential for detoxification of H_2_O_2_ produced by SOD activity and photorespiration.

Parallel enhancements of CAT and APX activities also have been expressed in *Centauries tuzgoluensis* under 150 mM NaCl stress treatments, but, at 300 mM NaCl, it was reported that CAT activity was increased, while APX activity remained unchanged. This suggests that CAT might be more important than APX in scavenging H_2_O_2_ during salinity stress [23]. The enzymes SOD, POD, CAT, and APX are important factors and suggest that they are correspondingly organized in relation to each other under saline environments (Table 2). The results showed that the models were significant at a 95% confidence limit and can be used to closely predict the values of the indices in the roots, as well as in the leaves of pistachio. Further studies on the localization of the models may require more validation, nevertheless, they were statistically significant in this study. The activity of enzymes and MDA content in the leaves of pistachio rootstock were positively correlated with those in the roots except between CAT and POD and all the measured values in both leaves and roots were significantly correlated to the saline water treatment regimes. Thus, it shows that the antioxidative responses of pistachio rootstock to salinity in both leaves and roots are cultivar dependent and have a relative adjustment mechanism.

### 3.4. Genetic Variation in Antioxidant Activity among Rootstock to Salt Stress

In this study, a significant correlation between the APX and GR activity in both leaves (0.95) and roots (0.96) were observed among the rootstock. Since APX and GR activities increased under salt stress, it is likely that the oxidative stress was efficiently reduced by the activity of the ascorbate–glutathione cycle spatially in the tolerant rootstock (UCB-1) followed by Badami and Ghazvini. In addition, the activity of GR in the roots, which is positively correlated with the other enzymes, suggests that non-enzymatic routes are as important as enzymatic routes for controlling the oxidative stress caused by salinity in pistachio rootstock. H_2_O_2_ is a toxic compound, which is produced due to significant changes in SOD activity [37]. Thus, GR content was significantly higher after NaCl treatment and was negatively correlated with SOD activity in roots (Figure 1 and Figure 5).

## 4. Materials and Methods

### 4.1. Plant Material and Growth Conditions

Four Iranian pistachio (*Pistacia vera* L.) rootstock (namely, Badami, Ghazvini, Akbari, and Kale-Ghouchi) and UCB-1, a hybrid (*P. atlantica* × *P. integerrima*) which has been extensively cultivated, and showing genetic variation for salt tolerance, were used in our studies (from this point on, Kale-Ghouchi will be abbreviated as KG). One-year-old uniform rootstock were prepared and transplanted to the 8 L greenhouse pots filled with sieved 2 mm sandy-loam soil (pH 7.86, EC 0.71 dSm^−1^, 0.84% organic, and 15% field capacity, available N, P, and K of 99.3, 29.6, and 289.4 mg kg^−1^, respectively).

### 4.2. Salinity Treatment and Sample Collection

After eight weeks of pre-culture, irrigation with four different concentrations of saline water (0.5 L per pot once in three days), with electrical conductivities (EC) including a control (0.42 dSm^−1^), 8 dSm^−1^, 12 dSm^−1^, and 16 dSm^−1^ were applied for 100 days. Salinity treatments were applied gradually to avoid osmotic shock. At three irrigations intervals, 200 mL of deionized water was applied to prevent salt accumulation. Greenhouse conditions were the following: temperature regime of 30/17 °C (day/night), with an average relative humidity of 75% and 14/10 h light/dark period at a photosynthetic photon flux density of about 400 to 500 µmol m^−2^ s^−1^. On days 0, 25, 50, 75, and 100 after the beginning of the salinity treatments, leaf samples were collected from each rootstock. Root samples were collected at day 100 after harvest and stored at −80 °C for further biochemical analysis. The MDA and antioxidant enzyme activity of SOD, POD, CAT, APX, and GR of both leaf and root samples were measured as described below.

### 4.3. Determination of Enzyme Activity

To determine the enzyme activity, both leaf and root samples were entirely milled using a cold mortar and pestle. A one-gram sample was homogenized in 5 mL of 50 mM sodium phosphate buffer (pH 7.8) and centrifuged at 13,000× *g* for 20 min at 4 °C. The supernatant was used to measure the enzyme activities.

#### 4.3.1. SOD Activity

To determine the SOD activity, the 3 mL reaction solution contained 13 mM methionine, 63 mM nitro blue tetrazolium chloride, 1.3 mM riboflavin, 50 mM phosphate buffer, and 50 mL of the enzyme extract [41]. The reaction mixture was incubated for 10 min and the absorbance was recorded at 560 nm. One unit of SOD activity corresponds to the amount of enzyme required for the inhibition of photochemical reduction of *p*-nitro blue tetrazolium chloride reduction by 50%.

#### 4.3.2. POD Activity

One gram of each leaf and root sample was separately milled in 5 mL of assay buffer. The homogenates were centrifuged at 12,000× *g* for 30 min at 4 °C [42]. Five mL of the assay buffer for the peroxidase activity contained the following: 125 μM of phosphate buffer, 50 μM of pyrogallol, 50 mM of H_2_O_2_, pH 6.8, and one mL of the 20 times diluted enzyme extract. This was incubated for 5 min at 25 °C and, subsequently, the reaction was stopped by adding 0.5 mL of 5% (*v*/*v*) H_2_SO_4_. The amount of purpurogallin was determined by measuring the absorbance at 420 nm.

#### 4.3.3. CAT Activity

To determine the CAT activity, the 3 mL reaction solution contained 15 mM H_2_O_2_, 50 mM phosphate buffer (pH 7.0), and 50 mL of the enzyme extract [43]. The reaction was initiated by the addition of the 100 μL enzyme extract, and the decrease in absorbance of H_2_O_2_ at 240 nm for 30 s was recorded.

#### 4.3.4. APX Activity

One gram of each sample was milled in 3 mL of extraction solution including 50 mM phosphate buffer (pH 7.0), 2 mM ascorbate, and 5 mM EDTA at 4 °C [44]. The suspension was centrifuged for 20 min at 13,000× *g*. The supernatant was used for analyzing the enzyme activity. The 3 mL reaction solution of APX contained 50 mM phosphate buffer (pH 7.0), 0.5 mM ascorbate, 0.1 mM H_2_O_2_, and 0.1 mL of enzyme extract. The reduction in the absorbance of ascorbate indicates the APX activity within 1 min at 290 nm. One unit of APX activity was defined as the amount of enzyme required for catalyzing the oxidation of 1 mmol ascorbate per minute.

#### 4.3.5. GR Activity

Extraction of GR was determined by measuring the reduction of GSSG by NADPH at 30 °C through the decrease in absorbance at 340 nm and via the extinction coefficient of 6.2 mM^−1^cm^−1^ [45]. The assay mixture contained 0.2 M potassium phosphate, 0.2 mM Na_2_EDTA, 1.5 mM MgCl_2_, 25 μM NADPH, 0.25 mM GSSH, pH 7.5, and 50 μL of enzyme extract in a 1 mL final volume. The reaction was initiated by the addition of NADPH.

### 4.4. Lipid Peroxidation

Lipid peroxidation was determined by measuring the amount of MDA produced by the thiobarbituric acid reactive substances assay. One gram of frozen leaf and root samples were milled separately in 5 mL of 1% trichloroacetic acid and centrifuged at 16,000× *g* for 10 min at 4 °C [46]. One mL of the supernatant was added to 4 mL of 20% trichloroacetic (0.5%). The mixture was heated at 95 °C for 30 min, subsequently chilled on ice for 30 min, and then the absorbance was measured at 450, 532, and 600 nm. The MDA concentration was calculated using the following equation:

MDA (μmol g^−1^ FW) = 6.45 × (OD_532_ − OD_600_) − 0.56 × OD_450_.

### 4.5. Statistical Analysis

A factorial experiment was done on the basis of randomized complete design (RCD) with three replications. All measurements were conducted by the analysis of variance (ANOVA) followed by Duncan’s new multiple range test [47], with PROC GLM of SAS (version 9.1, SAS Institute, Kerry, NC, USA). Pearson’s correlation coefficients were carried out using SAS software. The linear and quadratic regression models for the enzyme activities in both leaves and roots were evaluated using the coefficient of determination (R-square) to explain the variance of the data portrayed by the model.

## 5. Conclusions

This study demonstrates that low salinity stress (8 dS/m^−1^) does not impact rootstock but shows that there are significant increases in antioxidant enzymes and MDA content, particularly in susceptible genotypes. At moderate and higher salt levels (12 dSm^−1^ and 16 (dSm^−1^), all antioxidant enzymes, SOD, POD, and APX, significantly increased in leaves as well as roots. Pistachio root plays an important chromatic role in modulating the oxidative stress caused by salinity stress. Rootstock UCB-1 exhibited a better coordinated response to the salinity stress followed by Badami, Ghazvini, KG, and Akbari. The degree of variation in salinity tolerance is correlated to the cultivar ability to correspondingly organize the antioxidant enzymes at the root level or different ratios of antioxidant enzymes and MDA content across leaf cell membranes. This study suggests that the UCB-1 and Akbari are two contrasting cultivars to salinity stress and are well suited to use for further comparative salinity tolerance studies in pistachio.

## Figures and Tables

**Table 1:** Correlation analysis of superoxide dismutase (SOD), peroxidase (POD), catalase (CAT), malondialdehyde (MDA), ascorbate peroxidase (APX), and glutathione reductase (GR) content in the leaves and roots of five pistachio rootstocks.

Leaf	Leaf						Root					
	APX	CAT	POD	SOD	MDA	GR	APX	CAT	POD	SOD	MDA	GR
**APX**	1.00						0.99	0.98	0.83	0.97	−0.99	0.99
**CAT**	0.56	1.00					0.51	0.55	0.35	0.45	−0.56	0.58
**POD**	0.96	0.34	1.00				0.97	0.94	0.86	0.98	−0.95	0.94
**SOD**	0.85	0.28	0.91	1.00			0.88	0.81	0.96	0.93	−0.84	0.81
**MDA**	−0.99	−0.55	−0.95	−0.85	1.00		−0.96	−0.96	−0.86	−0.95	1	−0.99
**GR**	0.95	0.65	0.88	0.70	−0.94	1.00	0.91	0.90	0.72	0.89	−0.94	0.96
**Root**												
**APX**							1.00					
**CAT**							0.97	1.00				
**POD**							0.83	0.79	1.00			
**SOD**							0.98	0.93	0.88	1.00		
**MDA**							−0.98	−0.97	−0.83	−0.95	1.00	
**GR**							0.96	0.96	0.80	0.94	−0.99	1.00

The values more than 0.44 and 0.59 are significant at 0.05 and 0.01, respectively.

**Table 2:** Unbalanced completely randomized design between tolerant (UCB-1, Badami, and Ghazvini) and susceptible (Akbari and Kale-Ghouchi) rootstocks.

Source of Variation	df	Mean Square (Leaf)						Mean Square (Root)					
		APX	CAT	POD	SOD	MDA	GR	APX	CAT	POD	SOD	MDA	GR
**Group**	1	64.7 *	70	823.6 *	1127 **	0.02 *	0.007	11.71 *	12.19	205 **	358.4 *	0.01 *	0.025
**Error**	3	5.49	60	36.8	9.69	0.001	0.002	0.84	1.54	2.08	11.69	0.001	0.003

* and ** are 0.05 and 0.01, respectively.

## References

[1] Aliakbarkhani S.T., Farajpour M., Asadian A.H., Aalifar M., Ahmadi S., Akbari M. (2017). Variation of nutrients and antioxidant activity in seed and exocarp layer of some Persian pistachio genotypes. Ann. Agric. Sci..

[2] Munns R., Tester M. (2008). Mechanisms of salinity tolerance. Annu. Rev. Plant. Biol..

[3] Kurum R., Ulukapi K., Aydinskir K., Onus A.N. (2013). The influence of salinity on seedling growth of some pumpkin varieties used as rootstock. Notulae Bot. Horti. Agrobot. Cluj. Napoca.

[4] Hima Kumari P.S., Anil Kumar S., Sivan P., Katam R., Suravajhala P., Rao K.S., Varshney R.K., Kavi Kishor P.B. (2017). Overexpression of a plasma membrane bound Na+/H+-antiporter-like protein (SbNHXLP) confers salt tolerance and improves fruit yield in tomato by maintaining ion homeostasis. Front. Plant. Sci..

[5] de Azevedo Neto A.D., Prisco J.T., Enéas-Filho J., de Abreu C.E.B., Gomes-Filho E. (2006). Effect of salt stress on anti-oxidative enzymes and lipid peroxidation in leaves and roots of salt-tolerant and salt-sensitive maize genotypes. Environ. Exp. Bot..

[6] Gao S., Ouyang C., Wang S., Xu Y., Tang L., Chen F. (2008). Effects of salt stress on growth, antioxidant enzyme and phenylalanine ammonia-lyase activities in *Jatropa curcas* L. seedlings. Plant. Soil Environ..

[7] Blokhina O., Virolainen E., Fagerstedt K.V. (2003). Antioxidants, oxidative damage and oxygen deprivation stress: A review. Ann. Bot..

[8] Feng X., Lai Z., Lin Y., Lai G., Lian C. (2015). Genome-wide identification and characterization of the superoxide dismutase gene family in *Musa acuminata* cv. Tianbaojiao (AAA group). BMC Genom..

[9] Foyer C.H., Noctor G. (2011). Ascorbate and glutathione: The heart of the redox hub. Plant. Physiol..

[10] Wang Q., Wu C., Xie B., Liu Y., Cui J., Chen G., Zhang Y. (2012). Model analyzing the antioxidant responses of leaves and roots of switch grass to NaCl-salinity stress. Plant. Physiol. Biochem..

[11] Pandey H.C., Baig M.J., Chandra A., Bhatt R.K. (2012). Drought stress induced changes in lipid peroxidation and antioxidant system in genus Avena. J. Environ. Biol..

[12] Pandey S., Fartyal D., Agarwal A., Shukla T., James D., Kaul T., Reddy M.K. (2017). Abiotic stress tolerance in plants: Myriad roles of ascorbate peroxidase. Front. Plant. Sci..

[13] Begara-Morales J.C., Sánchez-Calvo B., Chaki M., Mata-Pérez C., Valderrama R., Padilla M.N., Barroso J.B. (2015). Differential molecular response of monodehydroascorbate reductase and glutathione reductase by nitration and S-nitrosylation. J. Exp. Bot..

[14] Mohammadi M.H.S., Etemadi N., Arab M.M., Aalifar M., Arab M., Pessarakli M. (2017). Molecular and physiological responses of Iranian Perennial ryegrass as affected by Trinexapac ethyl, Paclobutrazol and Abscisic acid under drought stress. Plant. Physiol. Biochem..

[15] Zhang M., Fang Y., Ji Y., Jiang Z., Wang L. (2013). Effects of salt stress on ion content, antioxidant enzymes and protein profile in different tissues of *Broussonetia papyrifera*. S. Afr. J. Bot..

[16] Walker R.R., Torokfalvy E., Behboudian M.H. (1987). Uptake; distribution of chloride, sodium and potassium ions and growth of salt-treated pistachio plants. Austral. J. Agric. Res..

[17] Aliakbarkhani S.T., Akbari M., Hassankhah A., Talaie A., Moghadam M.F. (2015). Phenotypic and genotypic variation in Iranian pistachios. J. Genet. Eng. Biotechnol..

[18] Van Breusegem F., Vranová E., Dat J.F., Inzé D. (2001). The role of active oxygen species in plant signal transduction. Plant. Sci..

[19] Chawla S., Jain S., Jain V. (2013). Salinity induced oxidative stress and antioxidant system in salt-tolerant and salt-sensitive cultivars of rice (*Oryza sativa* L.). J. Plant. Biochem. Biotechnol..

[20] Prashanth S.R., Sadhasivam V., Parida A. (2008). Over expression of cytosolic copper/zinc superoxide dismutase from a mangrove plant *Avicennia marina* in indica rice var Pusa Basmati-1 confers abiotic stress tolerance. Transgenic Res..

[21] Mittler R. (2002). Oxidative stress, antioxidants and stress tolerance. Trend Plant. Sci..

[22] Gill S.S., Tuteja N. (2010). Reactive oxygen species and antioxidant machinery in abiotic stress tolerance in crop plants. Plant. Physiol. Biochem..

[23] Yıldıztugay E., Sekmen A.H., Turkan I., Kucukoduk M. (2011). Elucidation of physiological and biochemical mechanisms of an endemic halophyte *Centaurea tuzgoluensis* under salt stress. Plant. Physiol. Biochem..

[24] Akbari M., Mahna N., Ramesh K., Bandehagh A., Mazzuca S. (2018). Ion homeostasis, osmoregulation, and physiological changes in the roots and leaves of pistachio rootstocks in response to salinity. Protoplasma.

[25] Zhang L., Ma H., Chen T., Pen J., Yu S., Zhao X. (2014). Morphological and physiological responses of cotton (*Gossypium hirsutum* L.) plants to salinity. PLoS ONE.

[26] Zhang Q., Zhang J.Z., Chow W.S., Sun L.L., Chen J.W., Chen Y.J., Peng C.L. (2011). The influence of low temperature on photosynthesis and antioxidant enzymes in sensitive banana and tolerant plantan (*Musa* sp.) cultivars. Photosynthetica.

[27] Etemadi N., Sheikh-Mohammadi M.H., Nikbakht A., Sabzalian M.R., Pessarakli M. (2015). Influence of trinexapac-ethyl in improving drought resistance of wheatgrass and tall fescue. Acta Physiol. Plant..

[28] Meloni D.A., Oliva M.A., Martinez C.A., Cambraia J. (2003). Photosynthesis and activity of superoxide dismutase, peroxidase and glutathione reductase in cotton under salt stress. Environ. Exp. Bot..

[29] Amor N.B., Jiménez A., Megdiche W., Lundqvist M., Sevilla F., Abdelly C. (2007). Kinetics of the anti-oxidant response to salinity in the halophyte *Cakile maritima*. J. Integr. Plant. Biol..

[30] Al-Taweel K., Iwaki T., Yabuta Y., Shigeoka S., Murata N., Wadano A. (2007). A bacterial transgene for catalase protects translation of D1 protein during exposure of salt-stressed tobacco leaves to strong light. Plant. Physiol..

[31] Azooz M.M., Ismail A.M., Elhamd M.A. (2009). Growth, lipid peroxidation and antioxidant enzyme activities as a selection criterion for the salt tolerance of maize cultivars grown under salinity stress. Int. J. Agric. Biol..

[32] Lin K.H., Pu S.F. (2010). Tissue-and genotype-specific ascorbate peroxidase expression in sweet potato in response to salt stress. Biol. Plant..

[33] Diaz-Vivancos P., Faize M., Barba-Espin G., Faize L., Petri C., Hernández J.A., Burgos L. (2013). Ectopic expression of cytosolic superoxide dismutase and ascorbate peroxidase leads to salt stress tolerance in transgenic plums. Plant. Biotechnol. J..

[34] Wang Y., Wisniewski M., Meilan R., Cui M., Webb R., Fuchigami L. (2005). Overexpression of cytosolic ascorbate peroxidase in tomato confers tolerance to chilling and salt stress. J. Am. Soc. Hortic. Sci..

[35] Demiral T., Türkan I. (2005). Comparative lipid peroxidation, antioxidant defense systems and proline content in roots of two rice cultivars differing in salt tolerance. Environ. Exp. Bot..

[36] Bandeoğlu E., Eyidoğan F., Yücel M., Öktem H.A. (2004). Antioxidant responses of shoots and roots of lentil to NaCl-salinity stress. Plant. Growth Regul..

[37] Hamed K.B., Castagna A., Salem E., Ranieri A., Abdelly C. (2007). Sea fennel (*Crithmum maritimum* L.) under salinity conditions: A comparison of leaf and root antioxidant responses. Plant. Growth Regul..

[38] Hediye Sekmen A., Türkan İ., Takio S. (2007). Differential responses of anti oxidative enzymes and lipid peroxidation to salt stress in salt-tolerant *Plantago maritima* and salt-sensitive *Plantago* Media. Physiol. Plant..

[39] Gharsallah C., Fakhfakh H., Grubb D., Gorsane F. (2016). Effect of salt stress on ion concentration, proline content, antioxidant enzyme activities and gene expression in tomato cultivars. AoB Plants.

[40] Cavalcanti F.R., Oliveira J.T.A., Martins-Miranda A.S., Viégas R.A., Silveira J.A.G. (2004). Superoxide dismutase, catalase and peroxidase activities do not confer protection against oxidative damage in salt-stressed cowpea leaves. New Phytol..

[41] Giannopolitis C.N., Ries S.K. (1977). Superoxide dismutases: I. Occurrence in higher plants. Plant. Physiol..

[42] Kar M., Mishra D. (1976). Catalase, peroxidase, and polyphenoloxidase activities during rice leaf senescence. Plant. Physiol..

[43] Cakmak I., Marschner H. (1992). Magnesium deficiency and high light intensity enhance activities of superoxide dismutase, ascorbate peroxidase, and glutathione reductase in bean leaves. Plant. Physiol..

[44] Nakano Y., Asada K. (1981). Hydrogen peroxide is scavenged by ascorbate-specific peroxidase in spinach chloroplasts. Plant. Cell Physiol..

[45] Sgherri C.L.M., Navari-Izzo F. (1995). Sunflower seedlings subjected to increasing water deficit stress: Oxidative stress and defense mechanisms. Physiol. Plant..

[46] Hodges D.M., DeLong J.M., Forney C.F., Prange R.K. (1999). Improving the thiobarbituric acid-reactive-substances assay for estimating lipid peroxidation in plant tissues containing anthocyanin and other interfering compounds. Planta.

[47] Duncan D.B. (1955). Multiple range and multiple F tests. Biometrics.

